# Prevalence of *Clostridium difficile* colonization among healthcare workers

**DOI:** 10.1186/1471-2334-13-459

**Published:** 2013-10-04

**Authors:** N Deborah Friedman, James Pollard, Douglas Stupart, Daniel R Knight, Masoomeh Khajehnoori, Elise K Davey, Louise Parry, Thomas V Riley

**Affiliations:** 1 Department of Medicine and Infectious Diseases, Barwon Health, Geelong, Victoria, Australia; 2 Department of Surgery, Barwon Health, Geelong, Victoria, Australia; 3 School of Pathology & Laboratory Medicine, The University of Western Australia, Crawley, WA 6009, Australia; 4 PathWest Laboratory Medicine, Queen Elizabeth II Medical Centre, Nedlands, WA 6009, Australia; 5 Deakin University Medical School, Geelong, Victoria, Australia

## Abstract

**Background:**

*Clostridium difficile* infection (CDI) has increased to epidemic proportions in recent years. The carriage of *C. difficile* among healthy adults and hospital inpatients has been established. We sought to determine whether *C. difficile* colonization exists among healthcare workers (HCWs) in our setting.

**Methods:**

A point prevalence study of stool colonization with *C. difficile* among doctors, nurses and allied health staff at a large regional teaching hospital in Geelong, Victoria. All participants completed a short questionnaire and all stool specimens were tested by Techlab® C.diff Quik Check enzyme immunoassay followed by enrichment culture.

**Results:**

Among 128 healthcare workers, 77% were female, of mean age 43 years, and the majority were nursing staff (73%). Nineteen HCWs (15%) reported diarrhoea, and 12 (9%) had taken antibiotics in the previous six weeks. Over 40% of participants reported having contact with a patient with known or suspected CDI in the 6 weeks before the stool was collected. *C. difficile* was not isolated from the stool of any participants.

**Conclusion:**

Although HCWs are at risk of asymptomatic carriage and could act as a reservoir for transmission in the hospital environment, with the use of a screening test and culture we were unable to identify *C. difficile* in the stool of our participants in a non-outbreak setting. This may reflect potential colonization resistance of the gut microbiota, or the success of infection prevention strategies at our institution.

## Background

*Clostridium difficile* infection (CDI) has increased to epidemic proportions in recent years [1,2]. It is estimated that approximately 20% of hospitalized adults are *C. difficile* carriers who shed the bacterium in their stool [3-5]. Clinical factors, such as previous *C. difficile*-associated disease and recent antibiotic use may be predictive of asymptomatic carriage [5].

The carriage of *C. difficile* has been studied among inpatients previously [6]. Inpatients at a large centre in Boston were sampled for the presence of *C. difficile* and 18% of admissions were positive for *C. difficile* at the time of admission, while a further 15% acquired the infection after testing negative on admission. Similarly, Johnson and others found that 21% of patients acquired *C. difficile* during hospital admission, and 85% of these patients were asymptomatic [7]. The risk of colonization increases at a steady rate during hospitalization, suggesting a cumulative daily risk of exposure to *C. difficile*, probably as spores, which can persist for months in the hospital environment [8,9].

Colonized individuals are by definition asymptomatic and not at greater risk of developing diarrhoea [6,7]. Indeed, colonization with non-toxigenic strains of *C. difficile* is protective against the development of disease with toxigenic strains [7]. However, it is possible that *C. difficile*-colonized individuals are a major source of nosocomial *C. difficile* contamination and infection [5,10].

In a variety of studies, the prevalence of *C. difficile* in the stool of healthy asymptomatic adults has varied from less than 2% up to 15% [8,11,12]. Carriage of a *C. difficile* strain among healthy adults may be either transient or persistent [11].

There are however several unanswered questions regarding CDI [13]. One of these includes the role of individuals colonized with *C. difficile* in the transmission of the organism in the healthcare setting. The carriage of *C. difficile* by healthcare workers (HCWs) has been studied previously with conflicting results. Carmelli and others cultured the stool of healthcare workers in a non-outbreak period, and found that none of 55 specimens contained *C. difficile*[14]. In a recent study in Austria, none of 112 HCWs was colonized with *C. difficile*[15]. However, in contrast, research in Japan and The Netherlands has found 4.3% and 13% of HCWs, respectively, were colonized with *C. difficile*[12,16].

It has been assumed that HCWs are at risk of asymptomatic carriage and that they too could act as a reservoir for transmission in the hospital environment. The hands of healthcare workers, transiently contaminated with *C. difficile* spores, are probably the main means by which the organism is spread during non-outbreak periods [8]. Strict adherence to hand washing with soap and water and glove use with changing of gloves between patient contacts, are the most effective ways to both prevent hand contamination with *C. difficile* and prevent the spread of spores [4,17,18].

Barwon Health is a large regional teaching hospital, with a total of 921 beds. No solid organ or bone marrow transplantation is performed at our institution. Prospective laboratory-based surveillance for CDI commenced in 2010, with cases being submitted to the Victorian Healthcare Associated Infection Surveillance System [19,20]. Rates of CDI at our institution in 2010 were 1.9 per 10,000 occupied bed days, 3 per 10,000 occupied bed days in 2011, and 2.4 per 10,000 occupied bed days in 2012, in keeping with the state aggregate in Victoria, Australia [20]. No seasonality of CDI had been noted at our institution.

This point prevalence study was performed in 2012 to determine the prevalence of *C. difficile* stool carriage among the major occupational groups of healthcare workers (HCWs) with significant patient contact in our medical setting.

## Methods

### Participant recruitment

HCWs from acute care, rehabilitation, and residential aged care at our institution were invited to participate over 14 weeks in 2012. Healthcare workers who were approached for participation included doctors, nurses, physiotherapists and occupational therapists. Participants were consented, and provided with a specimen collection kit and a questionnaire printed on their pathology form. Participants were identified by a unique study identifier number. There were no payments or incentives provided for participation in the study, and the Human Research and Ethics Committee at Barwon Health approved this research.

### Data collection

The pathology form included questions to ascertain basic demographic data and risk factors for *C. difficile* acquisition. Employment location was determined through tick boxes (Acute Hospital/Inpatient rehabilitation/Residential aged Care). Participants indicated their profession (Doctor/Nurse/Allied Health) as well as the type of workplace or ward (Aged Care/Emergency Department/Intensive Care Unit/Medical ward/Obstetrics/Paediatrics/Psychiatry/Rehabilitation/Surgical/Operating Theatre). Information was also sought about antibiotic therapy, contact with a patient with known or suspected CDI, and diarrhoea or hospitalization in the preceding 6 weeks. Participant study data identified by unique study number from the pathology request form was entered into the study database upon arrival in the laboratory.

### Microbiological methods

Faecal specimens were delivered to the microbiology laboratory identified only by study number. The Techlab® C.diff Quik Check Complete test kit (Blacksburg, VA), comprising a dual rapid membrane enzyme immunoassay for toxins A and B and for glutamate dehydrogenase (GDH) antigen, was utilized for initial screening of specimens.

In accordance with product recommendations, 25 μL of specimen was mixed to 750 μL of diluent and single drop of conjugate before transfer to the sample well. After incubation at room temperature for 15 min, the membrane was washed with 300 μL of wash buffer, and two drops of the substrate reagent was applied. The window, which contains an internal control, is read for up to 10 min whilst incubating at room temperature.

All specimens were subsequently frozen, then batched and forwarded to a reference laboratory in Western Australia for *C. difficile* culture. Attempts to isolate *C. difficile* were made based on previously described methods [21,22]. Stool samples were cultured on cycloserine cefoxitin fructose agar (CCFA) and in an enrichment broth containing gentamicin, cycloserine and cefoxitin (GCC broth). After 48 hours incubation, 1 mL of enrichment broth was alcohol shocked with an equal volume of absolute ethanol for 1 hour and then plated onto CCFA containing sodium taurocholate. All plates were incubated in an anaerobic chamber (Don Whitley Scientific Ltd., Shipley West Yorkshire, UK) at 37°C, in an atmosphere containing 80% nitrogen, 10% hydrogen and 10% carbon dioxide.

Putative colonies of *C. difficile* were identified on the basis of their characteristic odour (horse dung smell), colony morphology (yellow, ground glass appearance) and their chartreuse fluorescence under long-wave UV light (~360 nm). The identity of doubtful isolates was confirmed by Gram stain, a latex agglutination test kit (Oxoid Ltd., Basingstoke, Hampshire, UK) [23], and the presence of the L-proline aminopeptidase activity (Remel Inc., Lenexa, KS, USA).

### Data analysis

Non-identifiable data was entered into an excel spreadsheet. The continuous variable of age was described using the mean and range. Categorical variables were described using numbers and percentages.

## Results

A total of 214 healthcare workers were consented to participate in this research of whom 128 provided a stool specimen for analysis. The mean age of participants was 43 years (range 21–65 years), three-quarters were female, and most were nursing staff (Table 1). Stool provided was formed in the majority of participants, and 15% reported diarrhoea in the previous 6 weeks. Nearly half of the participants reported having contact with a patient with known or suspected CDI. One HCW of 128 (0.8%) tested positive by toxin and GDH assay but negative on culture. All other 127 samples tested negative by the screening test, and by culture.

**Table 1:** Characteristics of participating healthcare workers

**Characteristic**	**N (%)**
Female gender	99 (77)
Mean age (range) in years	43 (21–65)
Recent antibiotic use	12 (9)
Recent diarrhoea	19 (15)
Contact with *C. difficile* patient	53 (41)
Recent hospitalization	1 (0.8)
**Occupational group**	
Nursing staff	93 (73)
Medical staff	19 (15)
Allied health	16 (12)
**Stool consistency**	
Formed	86 (67)
Semi-formed	36 (28)
Unformed	6 (5)

## Discussion

So called hypervirulent strains of *C. difficile* have emerged internationally, and more recently in Australia, and are responsible for causing hospital outbreaks with high morbidity and mortality [2,20,24-26]. Between October 2010 and March 2011, 2.3% of *C. difficile* infections were caused by a hypervirulent strain in Victoria, Australia [20]. This burden of disease provides a good impetus to understand the likely sources of *C. difficile*.

Colonization with *C. difficile* is known to occur among patients, especially those with risk factors of previous CDI, recent antibiotic use and exposure to the healthcare environment [5,8]. Existing research on healthcare worker colonization with *C. difficile* has yielded conflicting results. Studies published in 1998 and 2012 did not detect *C. difficile* stool colonization among 55 and 112 healthcare workers, respectively [14,15]. In contrast, *C. difficile* carriage was detected in 4 of 30 HCWs (13%) by van Nood, and in 4.3% of hospital staff by Kato [12,16]. These data indicate that colonization among HCWs typically occurs at a similar frequency as colonization among healthy adults [8,11,12], and that a large occupational exposure would be required to result in colonization or infection among HCWs.

Arfons and others suggest that *C. difficile* may be an underappreciated occupational risk for healthcare workers. Many healthcare workers are immunocompromised or have chronic medical conditions, and antibiotic treatment in the preceding year has been reported by nearly half of healthcare workers [14,27]. In the present study although 9% of HCWs had taken antibiotics in the preceding six weeks, the mean age of participants was 43 years, and they are likely to represent a healthy adult population. Among healthy adults the innoculum of *C. difficile* required to result in detection in stool is large. Villano and others evaluated an oral suspension of nontoxigenic *C. difficile* in healthy adults and found that this nontoxigenic strain was found in the stool of subjects given 10^8^ spores twice daily [28]. The dose required for detecton in stool was still 10^4^ spores or more daily even when vancomycin was administered [28]. In addition, healthy adults not treated with antibiotics are likely to be protected from colonization with *C. difficile* by their indigenous faecal microbiota [29,30].

In the present study, healthcare workers including doctors, nurses, physiotherapists, and occupational therapists were approached for recruitment. These categories of healthcare workers were chosen because of their direct contact with patients. Our volunteers in this point prevalence study were predominantly female nurses as was found in Hell’s study [15]. Of all HCW groups, nursing staff are likely to have the closest contact with patients and be at greatest risk of exposure to stool.

The testing used in this study included the Techlab® C.diff Quik Check enzyme immunoassay followed by culture. Nucleic acid amplification tests can be as much as twice as sensitive as enzyme immunoassays and can detect *C. difficile* more accurately when used in populations with an appropriate pretest probability (i.e., patients with more than three unformed stools in a 24 hour period without an identified cause) [31]. Enrichment culture is more sensitive a method for the detection of *C. difficile* than nucleic acid amplification tests [32]. It was however unclear how these tests would perform in a population with low pretest probability of CDI. However, given that two microbiological methods, including enrichment culture, were used in our study, we believe the results are likely to be accurate.

There are limitations to this study. Firstly, it is possible that healthcare workers with heavy *C. difficile* exposure may have been unlikely to participate due to fear of identification. Therefore, the healthcare workers who provided a stool specimen may not have been a representative sample of HCWs and may have been biased towards those with no abdominal complaints, and therefore little *C. difficile* exposure. In addition, our study may have been underpowered to detect *C. difficile* colonization. Our initial study size calculation assumed a population of healthcare workers with an approximate *C. difficile* colonization rate of 10% and the resultant sample size was therefore estimated to be 130. Furthermore, given the stable (although increasing) incidence of CDI at our institution, our results may not be able to be extrapolated to other centres with a different staff mix, patient-mix, different use of antibiotics, and different rates of CDI.

## Conclusion

With the use of a screening test and culture, we were able to determine that colonization with *C. difficile* among HCWs in our setting is rare. This may reflect potential colonization resistance of the gut microbiota, or the success of infection prevention strategies such as hand hygiene and glove use at our institution.

## Abbreviations

C. difficile: *Clostridium difficile*; CDI: *Clostridium difficile* infection; HCW: Healthcare worker; GDH: Glutamate dehydrogenase; CCFA: Cycloserine cefoxitin fructose agar; GCC: Gentamicin, cycloserine cefoxitin.

## Competing interests

The authors declare that they have no competing interests.

## Authors’ contributions

DS and NDF conceived the study. NDF, JP, and DS participated in the design of the study. MK, EKD, and LP carried out participant recruitment, and participated in the coordination of the study. JP performed the screening tests on all stool via C.diff Quik Check Complete test kit. JP created and maintained a study database. DRK and TVR performed all stool cultures. NDF, LP, JP and EKD drafted the manuscript. All authors read and approved the final manuscript.

## Pre-publication history

The pre-publication history for this paper can be accessed here:

http://www.biomedcentral.com/1471-2334/13/459/prepub
